# Ending pandemics within the shadow of trade: reconciling equity in global health with coloniality*

**DOI:** 10.4337/cilj.2025.01.01

**Published:** 2025-06-01

**Authors:** Sharifah Sekalala

**Affiliations:** Global Health Law, University of Warwick

**Keywords:** global health, international trade, equity, colonialism

## Abstract

Inequities in access to life-saving medicines during pandemics are not aberrations but symptoms of the postcoloniality of global health law. This legal framework sustains the racialised exclusion of Global South people, preserving remnants of early global efforts to contain disease in colonial spaces in the pursuit of furthering global trade. Tracing the historical trajectory of access to medicines from racialised colonial efforts aimed at disease containment to the modern intellectual property regime reveals how global health law, as a postcolonial technology, reinforces racialised hierarchies and associated structural vulnerabilities, but may also offer emancipatory promises. But to what extent can this emancipatory promise provide a platform for transformative structural change in global health? Through the scholarship on reparative justice, this article argues that reparations are a necessary intervention to address inequities in global health. In order to be effective, reparations must go beyond acknowledging past injustices to instituting material changes in global health through new international agreements that centre the global south, reforming structures to enable vaccine manufacturing in the global south and greater south-south collaborations to tackle a culture of dependency.

## 1 Introduction

The enduring inequities in access to essential medicines, which have shaped the global response to pandemics from HIV/AIDS and Ebola to Covid-19, are best understood through the *longue durée* of colonial health governance and its legal entrenchment in contemporary global health systems. These disparities must be understood not as incidental to, but rather symptomatic of, a historically embedded legal architecture that has systemically privileged certain populations while rendering others structurally vulnerable. The exclusion of large swathes of the Global South from life-saving therapies is not a failure of international law but rather a continuation of colonial logics that once governed medicine as a mechanism of control, extraction, and racialised exclusion. These inequities have their origins in the colonial medical enterprise, which prioritised the health of colonial administrators, settlers, and military personnel while neglecting, and at times actively endangering, the well-being of colonised populations.^1^ This hierarchy, deeply racialised in its epistemology and practice, not only shaped the development of public health interventions but also structured early international health governance, particularly in the regulation of disease control and quarantine policies.^2^ These foundations persist today, most notably in the international intellectual property regime that continues to dictate production, distribution, and affordability of essential medicines. The Agreement on the Trade-Related Aspects of Intellectual Property Rights^3^ (TRIPS Agreement) exemplifies this legal continuity, granting multinational pharmaceutical corporations (mainly headquartered in the Global North) monopoly control over life-saving drugs, thereby excluding many in the Global South from equitable access to medical innovations, especially during pandemics.

While soft law legal interventions, such as the United Nations General Assembly (UNGA) Declarations of Commitment on HIV/AIDS,^4^ the Doha Declaration on the TRIPS Agreement and Public Health (Doha Declaration),^5^ and amendments to the TRIPS Agreement,^6^ have sought to introduce flexibilities permitting exceptions to allow access to medicines during crises, these measures have proven inadequate.^7^ Initiatives such as UNITAID and COVAX, which aimed to harness bulk purchasing to facilitate equitable distribution, ultimately fell short as structural measures that could be deployed in pandemics with serious medicine shortages. The pandemic starkly illustrated the enduring structural limitations of Global Health Law (GHL), which remains constrained by an essential contradiction of protecting health as a public good while safeguarding trade interests. This tension is not incidental; rather, it is an artefact of a colonial legal order that has historically prioritised economic power over collective health.

The implications of this history extend beyond the immediate health crises. As demonstrated by scholarship on the transatlantic slave trade’s enduring health effects, historical injustices have left indelible marks on contemporary global health outcomes.^8^ These material harms range from violence and lack of access to employment, health care, and education to enduring discrimination and racialisation.^9^ Studies have quantified the economic ramifications of these historical harms, estimating their material cost to be approximately USD 23 trillion.^10^

The question of reparations has increasingly gained traction in climate justice debates,^11^ yet GHL has thus far been neglected as a site for reparative intervention. I present a conceptual and practical model of reparative justice that accounts for the ways in which GHL has been complicit in the reproduction of structural inequalities and proposes legal pathways for meaningful restitution. A reparative justice account foregrounds the main ideas: (i) that reparations must acknowledge past injustices and their ongoing effects on affected communities; and (ii) reparations need to create a difference in people’s lives, whether materially, psychologically, legally, civically or politically.^12^ Therefore, reparations must go beyond financial assistance to be transformative and bring about the structural changes that are necessary for realising social justice, which is the only way to truly realise equity.

The rest of the article proceeds in two parts. Part II traces the historical links between colonial health governance, international law and contemporary access to medicines, illustrating how international legal frameworks have been instrumental in sustaining racialised health inequalities. This discussion is situated within postcolonial international legal theory, illuminating the postcolonial condition of international law, the structural design of international legal frameworks that enable the othering of postcolonial states, including through access to medicines. Part III conceptualises reparations as a legal and moral imperative for redressing colonial harms and examines their place within public international law and GHL, after which it articulates what reparations in GHL would entail, foregrounding access to medicines. Here, I articulate that a reparative justice framework necessitates an approach that moves beyond symbolic acknowledgement towards substantive redress. Reparations must be transformative, not merely compensatory, ensuring material, psychological, legal, civic, and political redress for affected communities.^13^ By foregrounding the necessity of reparations within GHL, I contend that redressing colonial injustices requires not only recognising their legal and historical impact on health governance but also articulating concrete mechanisms for structural transformation.

## 2 Global Health Coloniality And Access To Essential Medicines

Global discourse on the scarcity of essential medicines cannot be disentangled from the historical and enduring nature of coloniality. For postcolonial scholars, particularly those working within Third World Approaches to International Law (TWAIL), it is evident that the structural inequities in healthcare, pharmaceutical production, and disease control are products of deep-seated legal and political structures that date back to colonial rule and sustain present day injustices.^14^ Colonial-era notions of health, race, and governance have persisted into contemporary legal frameworks, facilitating ongoing disparities in access to essential medicines. This underscores the centrality of law in reproducing hegemonic power relations that privilege the Global North at the expense of the Global South.

### 2.1 Colonial medicine and the legalisation of discrimination

From the late nineteenth century onward, many colonial powers implemented what they deemed ‘colonial medicine’, ostensibly aimed at controlling diseases within colonised territories in ways that were highly extractive and sought to exert social control on the colonised.^15^ Authorities regularly dismissed indigenous healing practices as ‘primitive’ or ‘inferior’,^16^ thereby promoting Western medicine as the universal standard. Such medical policies served three interrelated functions.^17^

First, it portrayed local cultures and indigenous knowledge practices as inferior to justify the colonial project by implying that local populations were unable to manage their own health.^18^ Colonisers were deemed to be ‘civilising’ agents, bringing modern medicine to supposedly uncultured societies.^19^ This stance bolstered paternalistic ideologies of governance, portraying any violence or exploitation as an unfortunate but necessary cost in the broader mission of health improvement. Whether through forced vaccinations, quarantines, or non-consensual medical trials, colonial medicine functioned as a disciplinary mechanism, ensuring that the colonised were continually reminded of their subservience.

Second, racial inequalities were overtly codified in legal and regulatory structures of colonial medicine.^20^ Colonised subjects were often depicted as inherently diseased or prone to contagion.^21^ This framing legitimised draconian laws and policies ranging from the formation of a category of ‘native’ medicine which enabled the segregation of hospitals and strict quarantine laws that disproportionately targeted the colonised. Crucially, these arrangements did not terminate with the formal demise of colonial regimes; rather, they evolved into contemporary global health systems, continuing to marginalise large swathes of populations in the Global South.

Third, colonial governance was primarily concerned with preserving the health of workers critical to the colonial economy; often at the expense of broader public health measures for local communities.^22^ The viability of a colonial plantation or mining operation typically overshadowed any genuine concern for indigenous well-being. ^23^

Importantly, what these histories illuminate is that the governance of medicine and health was, from the outset, a legal-political issue. Legislation and administrative regulations codified racial segregation in hospitals or allowed for indefinite quarantine.^24^ The concept of ‘medical police’, prevalent in several empires, positioned local subjects as perpetual suspects of spreading disease, thereby necessitating intrusive governance in order to sustain extraction.^25^

### 2.2 Law, early global public health control measures and coercive border policing

Law has been central to the idea of global containment of disease since the early twentieth century. The development of trade routes linking Europe to its colonies precipitated anxieties about contagion. Imperial powers had dual imperatives: expand commerce through profitable global networks, but also insulate their territories from colonial diseases.^26^ This tension yielded thirteen international agreements (by the early twentieth century) aimed at harmonising and coordinating quarantine and health regulations.^27^

One key arrangement was the 1892 International Sanitary Convention, focusing exclusively on cholera and the sanitation measures required for shipping routes into Europe, particularly via the Suez Canal.^28^ The underlying fear was that India, seen as a hotbed of cholera, would transmit the disease to Europe through global shipping lanes.^29^ Similarly, the 1893 International Sanitary Convention mandated notification procedures for cholera outbreaks, granting legal sanction to a set of exclusions designed to keep ‘infected’ populations at bay.^30^ These regimes were framed as technical or scientific endeavours, yet they were deeply influenced by prejudices that associated non-White bodies with contagion and social disorder.^31^

These early international agreements on health exemplify the postcoloniality of international law, buttressing hierarchical global orders, and can be read as what Makau wa Mutua describes as discursive and legal technologies that disciplined the ‘other’, by recasting entire peoples as disease carriers who must be contained for the greater good of global trade.^32^ Hence, these regimes set the stage for modern global health governance, where the health of the colonised was subsumed within concerns for trade to ensure accumulation.

Modern States have also continued the practice of border closures as a reflex response during pandemics or outbreaks, despite the World Health Organization (WHO) cautioning that such measures often have limited epidemiological benefit.^33^ Crucially, these closures primarily target countries in the Global South, reinforcing stereotypes that conflate non-Western populations with disease. A salient illustration is the use of discriminatory border closers in the Covid-19 pandemic, reifying the idea of the border as a legal site for exclusion of the foreign ‘other’. Despite the high Covid-19 case rates in the United States of America (USA),^34^ the United Kingdom (UK) did not impose similar travel bans on USA travellers, even though it had previously expressed scepticism about border closures, citing concerns about trade.^35^ Similarly, South Africa’s role in sequencing and sharing the Omicron variant with the global community, was met with punitive border closures by European and North American countries, ironically punishing South Africa, the very State that contributed significantly to global scientific knowledge.^36^ These measures vividly recall colonial ‘sanitation cordons’, designed to keep the imperial centre safe while undermining any reciprocal sense of solidarity.^37^ Equally striking was the rapid decision by nations of the Global North to sever ties not just with South Africa but in many cases with all African countries following South Africa’s genomic sequencing of the Omicron variant.^38^ This was particularly problematic because later sequencing illustrated that the variant had already been circulating in Europe.^39^

Still, border closures are not limited to North-South relations. During the 2014–2016 Ebola crisis in West Africa, numerous African countries shut their borders to neighbouring States without confirmed cases.^40^ This form of ‘coercive control’ has strong colonial echoes, further normalising the notion that certain bodies (predominantly those of colour) pose a universal biosecurity threat. Meanwhile, flows of samples and research teams from West Africa to Europe and North America were often permitted, underlining the economic prerogatives that remain deeply entwined with these policies.^41^ Through these African border closures, we can see the inherent postcoloniality that animates the operation of international law, where even postcolonial States reproduce coercion, often, through a misguided belief that this will insulate them from travel bans and protect their trade interests.

What these practices represent is the continuity in the structural violence that animates global health governance. TWAIL scholar Bhupinder S Chimni has long argued that contemporary international law often rationalises power asymmetries through purportedly neutral norms.^42^ Whether via quarantine laws or contemporary travel bans, law becomes the instrument of selective inclusion and exclusion, reinforcing the subordination of the Global South under a veneer of scientific logic.

### 2.3 Reproducing inequalities through the political economy of pharmaceutical production within the multilateral order

#### 2.3.1 Extracting raw materials from the global south

During the colonial era, certain regions of the Global South became sites for extracting raw materials crucial to drug manufacturing.^43^ These territories often housed botanical or mineral resources essential for creating pharmaceutical products used both locally and in metropolitan centres. European (and, later, American) corporations built subsidiary operations in colonised zones, exploiting the cheap labour and raw materials yet precluding any meaningful transfer of knowledge or technology.^44^ However, such arrangements were designed to suppress industrial development, ensuring that colonies remained dependent on imports for medical supplies, including pharmaceuticals. Even when pharmaceuticals were being produced, countries in the Global South rarely had comprehensive industries and largely created filler drugs that were incapable of sustaining a self-sufficient pharmaceutical sector. This allowed production and research to remain concentrated in Europe and later in the USA.^45^

The transition to post-independence did not substantially dismantle these extractive structures. Several African States, for instance, attempted to establish domestic pharmaceutical firms in the early postcolonial phase.^46^ Yet, these were frequently hamstrung by a confluence of factors: insufficient capital investment, reliance on imported machinery, and little State capacity for research and development.^47^ These nations faced significant structural barriers in developing their own pharmaceutical production capacity. Geoffrey Banda, Samuel Wangwe and Maureen Mackintosh argue that while some pharmaceutical industries did exist in the early post-colonial period, many were State-run or heavily dependent on foreign technology and investment.^48^ Many of these pharmaceutical industries collapsed in the 1980s and 1990s due to the Washington Consensus which led to a multilateral trading system that frowned against national protection for infant industries and introduced tighter rules on State aid in order to ostensibly level the playing field for global goods and services.^49^ The Washington Consensus refer to a list of ten economic reforms promoted by the International Monetary Fund, World Bank, and US Treasury in the 1980s and 1990s, encouraging trade liberalization and privatization of state-owned enterprises and other neoliberal reforms, especially in developing countries, primarily Latin America.^50^ These policies dismantled protectionist measures that had initially supported domestic pharmaceutical industries, leading to a dominance of multinational corporations in African medicine markets for instance.^51^

### 2.4 Experimenting on ‘other’ bodies from the global south

#### 2.4.1 Colonial precedents for unethical experimentation

The exploitative character of colonial medicine did not merely revolve around resource extraction or quarantines; colonised peoples were frequently subjected to clinical trials without consent. The impetus behind this was twofold: the colonised were considered readily available test populations, and the constraints of medical ethics were rarely applied to them. The infamous Tuskegee Syphilis Study (1932–1972) in the USA, which deliberately withheld treatment from African American men to observe the progression of untreated syphilis, was directly influenced by earlier colonial medical experiments conducted in the Philippines, Algeria, and India.^52^ Each of these experiments was buttressed by legal apparatuses that shielded institutions and practitioners from accountability.^53^ Whether through colonial ordinances authorising medical surveillance or domestic laws in the USA that disregarded informed consent, the pattern was consistent: racialised populations were rendered legally and morally expendable. The birth of modern bioethics through modern institutional review boards (IRBs) tasked with enforcing informed consent or international guidelines like the Declaration of Helsinki emerged only after public outcry over such abuses which underscores how structural power shapes the definition and enforcement of ethical standards.^54^

#### 2.4.2 Contemporary outsourcing of clinical trials

In the contemporary era, as it became increasingly difficult to experiment in the Global North, clinical trial research has been outsourced abroad to sites in the Global South to ensure that clinical trials could scale up.^55^ In many of these sites, pharmaceutical companies rely on much weaker standards. Large pharmaceutical corporations often situate clinical trials in the Global South to reduce costs and expedite approvals.^56^ Regulators in wealthy nations accept data from these trials, yet local participants rarely benefit from guaranteed treatment or affordable drug pricing once the products reach the market.^57^ In effect, new knowledge is extracted from the Global South, while the resulting innovations remain locked behind patent protections and commercial trade secrets in the North. Meanwhile, the participants themselves, hailing from vulnerable communities, are often left with negligible recourse if experiments cause harm. In Uganda, for instance, clinical trials for anti-retroviral drugs continued to put patients on a placebo and were not given the drug even after the clinical trial had shown that these drugs were effective.^58^ In effect this means that although the Global South has remained an important site for experimentation it does not fully share in the proceeds of new drugs, as these are still subject to patents that are bound up in an extractive intellectual property regime.

### 2.5 Restricting local pharmaceutical production in the Global South

A major factor limiting pharmaceutical production in Africa and other parts of the Global South is the intellectual property regime, particularly the TRIPS Agreement enforced by the World Trade Organization (WTO). The patent system continues to favour pharmaceutical companies based in the Global North, allowing them to maintain long-term monopolies over essential medicines while restricting local production in the Global South.^59^ Crucially, the enforcement of strict patent protections has inhibited the development of generic medicine production in African countries, despite the clear need for affordable medicines to address public health crises.^60^

Modern intellectual property law’s foundations can be traced to colonial concepts of property.^61^ The property regimes that once facilitated land expropriation, forced labour, and resource extraction were easily adapted to protect knowledge and innovation forms deemed lucrative for Western corporations. Amaka Vanni’s analysis is particularly illuminating, as she contends that a historical examination of the contemporary patent regime shows how colonialism, racism and inequality became deeply sedimented into the international intellectual property law, particularly patents, to enforce a particular type of property rights and to protect the economic interest of the transnational capitalist class.^62^ This assertion underscores how the modern intellectual property system continues to serve as an instrument of economic imperialism, whereby the commodification of life-saving medicines is not simply an unintended consequence, but a deliberate outcome designed to maintain the lucrative monopolies of pharmaceutical corporations. Consequently, the transformation of essential medicines into market commodities is intrinsically linked to the legacies of colonial extraction and the subsequent racial capitalist logics that define global trade relations, thus perpetuating a system in which the health needs of populations in the Global South are subordinated to the profit imperatives of the Global North. As such, the enforcement of strict intellectual property regimes is inextricably linked to the preservation of corporate profits, a mechanism that operates to the detriment of public health in the Global South. The consequent commodification of healthcare is emblematic of a broader system of racial capitalism in which the accumulation of capital is predicated upon the exploitation and devaluation of non-white lives, thereby perpetuating an inequitable global order that is as much legal as it is economic.^63^

### 2.6 Vaccine apartheid and the limitations of global health law

By late 2022, many states in the Global North had inoculated more than 80% of their populations, while vast swathes of the Global South were struggling to reach a 25% vaccination threshold.^64^ Activists described this profound disparity as ‘vaccine apartheid’, underscoring the structural violence that underlies global health inequities.^65^ The term’s invocation of apartheid was not accidental; rather, it reflected the argument that, like South Africa’s system of legislated racial subjugation, these inequities were the predictable outgrowth of entrenched colonial power asymmetries. Legal, economic, and institutional mechanisms, particularly intellectual property laws, maintain the Global South in a state of dependency, preventing equitable access to life-saving medicines. Just as apartheid employed legal measures to divide resources, healthcare, and mobility along racial lines, intellectual property laws depend on a similarly exclusionary logic, permitting wealthier and more politically powerful states to determine who is granted access to life-saving treatments.^66^

Two significant factors undergird these ongoing inequalities. Firstly, the much-touted TRIPS ‘flexibilities’ frequently fail to address the real needs of Global South States.^67^ For instance, proposals to waive patent rights for Covid-19 vaccines, championed by South Africa and India, faced intransigent opposition from key players, including the USA, the European Union (EU), and the UK.^68^ This resistance, whether motivated by self-interest or the desire to safeguard pharmaceutical industries interests, effectively nullified the possibility of manufacturing vaccines in the Global South. When Global South States have invoked compulsory licensing, they encounter considerable external pressure, as illustrated by Colombia’s experience with antiretroviral HIV/AIDS medication for which it remains entangled in ongoing litigation with the drug’s patent holder.^69^ Moreover, the complexities of mRNA technology further limited the efficacy of TRIPS’ flexibilities by rendering reverse-engineering efforts impracticable without substantial technology transfer – assistance that remained under the purview of pharmaceutical conglomerates in the Global North.^70^

Secondly, philanthropic or donor-based strategies, exemplified by initiatives like COVAX, proved insufficient in redressing these disparities.^71^ Although ostensibly designed to foster equitable vaccine distribution through pooled procurement, many wealthy governments opted to hoard vaccines or merely donate surpluses, rather than commit fully to collective bulk-purchasing and equitable allocation.^72^ The resultant shortfalls left Global South States beholden to ad hoc donations or bilateral agreements, effectively mirroring patterns of colonial paternalism.^73^ Even global funding agencies, such as the EU, which allocated resources for vaccine production initiatives, rarely addressed the more fundamental requirement of technology transfer to countries in the Global South which would have enabled them to make vaccines themselves.^74^ Consequently, substantial pledges of financial support were unable to yield meaningful local production when patent protections and legal barriers to manufacturing remained intact.

In the aftermath of the Covid-19-pandemic, it has become apparent that the structural logic of vaccine apartheid is not merely an economic phenomenon but a broader manifestation of racial capitalism, wherein the residual legacies of colonialism shape legal frameworks, trade policies, and technology flows.^75^ Scholars such as Achille Mbembe underline how this entrenched hierarchy results in the systematic exposure of certain populations – predominantly in the Global South – to heightened vulnerability, ill health, and even premature death.^76^ While the WHO has proposed reverse-engineering mRNA vaccines and assisting countries in establishing domestic vaccine production,^77^ these measures remain undermined by the absence of robust legal and political mechanisms to ensure technology transfer. Reliance on external funding programmes that impose stringent conditions often delays or constrains domestic vaccine manufacturing capacity.

Ultimately, the phenomenon of vaccine apartheid stands as a stark indictment of GHL’s deficiencies in balancing public health with entrenched commercial interests. As long as intellectual property frameworks remain tailored to safeguarding corporate profits in the Global North, and philanthropic approaches continue to place the Global South in a position of perpetual reliance, the fundamental inequities of the global health architecture will persist – rendering future pandemics and health crises more likely.

### 2.7 The postcoloniality of international intellectual property law

The persistence of colonial legacies in the TRIPS regime reveals a historical continuum whereby legal frameworks operates mechanisms that systematically entrench inequalities in access to essential medicines. The regime which is at the heart of sustaining pharmaceutical manufacturing in the Global North is not sustainable without reproducing hierarchies, meaning that some countries, predominantly postcolonial States, are perpetually doomed to be customers for drugs produced from elsewhere.

The globalisation of property rights has led to the material dispossession of formerly colonised people.^78^ This form of dispossession was crucial to the formation of international law.^79^ That the impetus for TRIPS negotiations was the threat posed by increasingly strong pharmaceutical industries in the Global South like Brazil and India, which had been protected much in the same way as many industries in the Global North had been in their infancy.^80^ In response to this threat, pharmaceutical companies pushed for the inclusion of intellectual property rights in the TRIPS Agreement as part of a broader package of international economic agreements within the WTO, illustrating how the coercive nature of international law negotiations reinforced the unequal bargaining asymmetries at the core of WTO negotiations.^81^ Far from being a peripheral concern, the issue of intellectual property rights is deeply interwoven with broader discourses on sovereignty and development. The TRIPS regime illuminates that so-called ‘sovereign’ States in the Global South are constrained in their capacity to formulate independent health and industrial policies.^82^ This scenario is emblematic of what Pahuja terms ‘tradeled development’:^83^ a model that privileges the free flow of capital and goods while curtailing States’ regulatory autonomy to safeguard public health, a continuation of the post-colonial era restrictions on industrial development in formerly colonised territories.^84^

Without dismantling the neocolonial structures that govern access to medicine, future pandemics will only replicate the same injustices, ensuring that pharmaceutical sovereignty remains concentrated in the Global North while the Global South continues to suffer disproportionately from preventable disease and death.^85^ Vaccine apartheid is thus not merely a crisis of Covid-19, but a symptom of a much deeper historical and structural problem, one that demands decolonisation of global health systems.

## 3 Reparations

### 3.1 Conceptual foundations of reparations

Reparations, originally derived from the word ‘repair’, refers to the process and result of acknowledging and remedying the damage or harm caused by an unlawful act.^86^ The idea of reparation, of amends owed for wrongs and wrongful harms, is ancient, universal, and a basic intuition of justice.^87^ More recently, the High Commissioner for Human Rights, defined reparations as actions that redress human rights violations by providing non-material or material benefits to the affected individuals or groups.^88^

There is an interdisciplinary literature on reparations. A lot of the earlier literature focused on remedying the longstanding impacts of the transatlantic slave trade.^89^ Increasingly, however, the scope of research on reparations has expanded to include reparations for indigenous peoples’ rights,^90^ colonialism,^91^ war crimes and genocide^92^ and increasingly ongoing events such as environmental justice and climate harms and the enduring harms of cultural or historical events.^93^

Reparations were initially used at the individual level to enable those whose rights had been violated to make claims against the State or non-State individuals.^94^ Following the end of World War II through the Nuremberg and Tokyo trials, the international community attempted to formulate ideas of collective justice through ideas of remedial justice in national and international contexts.^95^ The customary forms through which reparations are conceived, were subsequently codified in the UN’s *Basic Principles and Guidelines on the Right to a Remedy and Reparation for Victims of Gross Violations of International Human Rights Law and Serious Violations of International Humanitarian Law* (Basic Principles). The Basic Principles codified various forms of reparations which have largely been used after humanitarian crises as restitution, compensation, rehabilitation, satisfaction, and guarantees of non-repetition.^96^

In a 2020 report, the UN Special Rapporteur on Racism, Racial Discrimination, Xenophobia and Related Intolerance highlighted the need for reparatory measures that prompt substantive structural reforms, emphasising how the enduring legacies of slavery and colonialism contribute to current forms of structural racism and demands for reparative justice.^97^ In particular, the Rapporteur noted that colonial-era institutional frameworks persist in reproducing and intensifying longstanding inequalities.^98^ A parallel view was offered by the Group of Experts on People of African Descent, who explicitly connected the harms stemming from the transatlantic slave trade to ongoing contemporary injustices.^99^ Such arguments are situated within a broader decolonial tradition that locates the *longue durée* of slavery and colonialism in reparative thought and calls for structural change.^100^

Effective reparation programmes encompass both retrospective and prospective dimensions.^101^ The retrospective dimension addresses historical injustices, offering redress and compensation to those harmed by past wrongs. Meanwhile, the prospective dimension looks towards the future, focusing on policies and practices that dismantle the structural inequalities ensuring that the structural conditions that allowed for past injustices are actively dismantled, thereby averting future harms.^102^

### 3.2 Reparations in global health law

A central question arises when tracing a colonial genealogy of GHL’s formation and its impact on access to medicines: can one build a credible argument for reparations under international law based on these colonial origins? If so, what might constitute the legal and conceptual underpinnings of such a claim? Scholars working in the TWAIL tradition have consistently maintained that the legacies of slavery, colonialism, and subordination fundamentally shape international law to the detriment of States in the Global South.^103^ These scholars likewise argue that ‘western reparations’ are already woven into international legal and economic regimes, serving to benefit States in the Global North through global capital networks, extractive sovereign debt structures, and treaties that enshrine property rights at the expense of other interests.^104^ By contrast, attempts by formerly colonised States to articulate demands for reparations often encounter resistance within these same legal frameworks. Nevertheless, indications exist that international law could recognise or respond to reparations claims for historical colonial harm.^105^

### 3.3 Legal obstacles to reparations

Those attempting to use international law for reparations have faced three legal obstacles. The first is the issue of causation: Can historical colonial wrongs still be seen as causing present-day harms, given the passage of time and the possibility of mitigating factors, some of which may have been caused by post-colonial governments themselves?^106^ Secondly, the intertemporal doctrine holds that acts should be assessed by the legal standards prevailing at the time they occurred.^107^ This raises the dilemma of whether (if colonial subjugation was formally legal under historical norms) liability for colonial-era wrongdoing could be legally recognised at all. Lastly, the question of corporate liability emerges as international law typically defines States as primary subjects, leaving multinational corporations (which often reap substantial benefits from contemporary forms of economic and health-related exploitation) beyond direct legal accountability.^108^

Recent scholarship, however, has begun to propose legal theories and interpretive approaches aimed at addressing these hurdles. Henning Grosse Ruse-Khan and Ashrutha Rai, for instance, emphasise a focus on ongoing harm rather than purely historical counterfactuals, contending that accusations of postcolonial government inefficiency do not negate the fundamental colonial basis for harm; and thus the potential for reparations.^109^ As regards the intertemporal doctrine, the International Court of Justice’s (ICJ) reasoning in the advisory opinion on the *Legal Consequences of the Separation of the Chagos Archipelago from Mauritius in 1965*^110^ offers a precedent wherein the ICJ rejected the view that a right must be codified in law at the precise time of the initial violation in order to be invoked subsequently.^111^ Moreover, certain human rights bodies like the Inter-American Court of Human Rights have similarly found that invoking temporal jurisdiction to deny claims can perpetuate ongoing violations.^112^ Finally, while corporate subjecthood in international law remains unsettled, scholarship has increasingly questioned whether the human rights obligations of multinational enterprises (and the investment protections they enjoy) necessitate a rethinking of accountability frameworks when corporations profit from structural harms inherited from colonial rule.^113^

### 3.4 Reparations in international human rights law

Certain domains of international law, such as human rights law or transitional justice frameworks, have historically proven more amenable to the concept of reparations.^114^ For instance, reparations mechanisms have been implemented in the aftermath of armed conflict or serious human rights violations, often underpinned by instruments and jurisprudence that acknowledge State responsibility and victim redress. The Truth and Reconciliation Committee of Sierra Leone, for instance, recommended a comprehensive reparations program for victims of the 1991–2002 armed conflict, that included extensive health measures such as free physical and mental health for amputees and victims of sexual violence, as well as broader social economic rights for those whose health had been impacted through monthly pensions, education programs and microcredit projects for the severely wounded.^115^ However, despite the establishment of the Sierra Leone Reparations program, only financial aid through cash grants, minimal medical interventions, vocational training and symbolic measures were effected due to lack of funding.^116^

By contrast, achieving reparations specifically for global health harms poses unique legal and practical hurdles. While humanitarian law and transitional justice processes might integrate certain forms of medical assistance or rehabilitation, there is comparatively less precedent for reparations that address ongoing structural inequities in global health.^117^ As a result, health-related claims have seldom gained traction as explicitly ‘reparative’, which suggests that establishing direct legal and conceptual pathways for health reparations is a pressing gap in international law. Including health considerations in future reparations frameworks would necessitate both recognising the specific ways global health inequities evolve and devising enforceable standards capable of compelling structural change, an approach that conventional reparations programmes have not consistently delivered.^118^

### 3.5 Reparations, health and human rights

Health and human rights scholars contend that the right to health stands at the heart of GHL.^119^ Accordingly, approaching reparations through a human rights framework could prove fruitful, particularly as numerous scholars have conceptualised the worldwide inequity of access to medicines as a form of human rights violations.^120^ Under human rights law, as a constructed legal system, reparations function as a formal means of redressing harm arising from infringements. Felix Torres, for example, underscores the significance of the international human rights regime by emphasising its focus on the inherent dignity and worth of each individual; a principle that not only undergirds the moral rationale for reparations but also drives corrective justice in situations of human rights breaches.^121^ In this context, reparations provide a tangible set of measures aimed at rectifying infringements and affording victims some measure of restitution.

However, Torres’ understanding of reparations presupposes a relationship between the State the individual.^122^ This perspective is especially relevant in that it informs how one might conceptualise economic, social and cultural rights, such as the right to health, in line with the recommendations of the Committee on Economic, Social and Cultural Rights (CESCR). In cases of violence, the CESCR has emphasised the need for public authorities to enhance the socio-economic programmes to increase the number of people enjoying their economic, social and cultural rights.^123^

To situate reparations beyond the national sphere and thereby connect colonial-era injustices to a human rights-based framework, one must consider broader systems of redistribution, which in human rights discourse derive from the obligation of international assistance in the International Covenant on Economic Social and Cultural Rights (ICESCR).^124^ Article 2(1) of the ICESCR enjoins states, both individually and ‘through international assistance and co-operation’ to fulfil their obligations under the treaty, including the realisation of the right to the highest attainable standard of health.^125^ This requirement has been expounded through various General Comments, notably General Comment No. 14, which clarifies that sates carry a responsibility to support other states in fulfilling the right to health; an interpretation that the CESCR has repeatedly affirmed.^126^

Such a human rights-based approach to a ‘duty to assist’ aligns with wider theories of redistributive justice, as articulated by Elise Klein and Elizaveta Fouksman who posit that individuals who have suffered historical violations should be integrated into larger redistributive frameworks designed to restructure unjust socio-economic conditions while enabling ‘recognition and truth telling.’^127^ This vision encompasses both the formal acknowledgement, already evident in certain human rights documents that link prior transgressions with socio-economic fragility affecting parts of the Global South,^128^ together with an explicit obligation for responsible states to direct resources towards strengthening the socio-economic systems of less privileged states. From this perspective of redistributive justice, authentic justice remains incomplete in the absence of reparations. Yet, just as with many human rights standards, the precise meaning of implementing this obligation remains ambiguous in current practice.^129^

### 3.6 Reparations in practice

Although these objections are not insurmountable, apart from the UN reports which have contributed to the idea of symbolic reparations,^130^ there have been very few attempts at implementing such reparations. For instance, in 2022, the Dutch Prime Minister, Mark Rutte, offered a formal apology on behalf of the Netherlands for its role in enslaving approximately 600,000 people from Africa and Asia through the transatlantic slave trade during the 17th and 18th Centuries.^131^ Alongside the apology, the Dutch government pledged to spend EUR 200 million on awareness projects and a further EUR 27 million to establish a slavery museum.^132^

Similarly the German government has apologised for colonialism in Tanzania,^133^ and the Danish government has also issued an apology for its participation in the transatlantic slave trade.^134^ In the context of the UK, King Charles III expressed personal and profound sorrow for Britain’s role in the transatlantic slave trade while giving speeches in Rwanda and Jamaica in 2024.^135^ However, the British government has been reluctant to give any official apology or recognition of the UK’s role in the slave trade.^136^

Attempts to achieve material reparations which involve forms of compensation have been challenging to realise. For example, in 2021, Germany acknowledged having committed genocide during its colonial rule of Namibia and agreed to pay EUR 1.1 billion in financial aid.^137^ However, the agreement avoided using the terminology of reparations and framed the payment as aid instead.^138^ Such measures, when framed as aid rather than reparations, makes it dubious whether this can truly constitute reparative justice.

With respect to health, reparative efforts on slavery acknowledge the intergenerational health harms that continue to impact the descendants of slavery with worse health outcomes than their white counterparts in the USA.^139^ These reparative strategies have focused on structural changes to three areas of harm affecting black communities in the USA: discrimination against black doctors in training, large scale medical harms inflicted on particular groups such as Tuskegee experiments,^140^ and ongoing intersectional harm as is evident in the significantly higher mortality rate for black women compared to their white counterparts.^141^ In response, attempts at reparations have been mixed. Symbolic reparations in the form of acknowledgement of racialised harms, such as the American Medical Association which was founded in 1847, which finally acknowledged in 2008 its historical role in the segregation of healthcare in the USA and the exclusion of black physicians.^142^ However other attempts, for instance to establish trusts that structurally respond to persisting inequities in black women, remain in their conceptual stages.^143^

While colonial practices yield enduring inequities in access to essential medicines, one can argue that international law’s existing normative commitments in fields such as human rights and global health impose continuing duties. Though legal doctrines like causation, intertemporal law, and the limited liability of corporations pose formidable difficulties, emerging analyses suggest that these doctrines might be interpreted or revised to accommodate reparative claims.^144^ Whether such arguments can gain traction in practice remains uncertain; particularly in view of the intense political opposition faced by States and communities who seek restitution for colonial injustices. Yet, these theoretical openings reflect a growing acknowledgment that reparation for colonial-era harm may be not only legally plausible but morally and structurally imperative, especially as inequities in public health continue to expose the legacies of imperial extraction.

## 4 Towards A ‘Reimagined Reparatory Approach’ In Global Health Law

To reimagine and strengthen the concept of health reparations, I propose a more comprehensive approach that addresses the deep-rooted colonial systemic inequities embedded in the global access to medicines, which perpetuate continuous harm. My vision builds on Olufemi Táíwò’s ideas, which conceptualise reparations as a tool within a broader worldmaking project to create a more just and equitable system.^145^ Táíwò criticises certain forms of reparations such as symbolic reparations and public apologies, arguing that these can be exploited as they are often the cheapest forms of reparations.^146^ His account is grounded in the understanding that if unjust systems remain in existence and continue to reproduce themselves, they will perpetuate harm for future generations.^147^

There is an inherent contradiction in trying to seek reparations within Global Health Law after acknowledging the colonial system that underlines it. For some scholars nothing short of a total change of systems can ever lead to the structural and material changes that are necessary.^148^ However, it is possible to critique the international law system while also strategically advocating for its reform.^149^ Taiwo’s conception allows us to acknowledge that the reparations as part of redistributive justice will always be a gradual struggle that utilises different forms of struggle ie; political, legal and grassroots struggles. This is particularly important with a problem like access to medicines where any structural changes must account for the material realities of those who need access to medicines. For instance, while we may think that programs like donor programs such as COVAX/UNITAID are colonial in nature, they still have saved people’s lives. Therefore, it is important to acknowledge that a focus on changing international law through for instance calling for countries to walk away from the TRIPS Agreement is unlikely to be successful and efforts to create greater access to medicines through international law will be only be a partial part of broader struggles.

Drawing on Taiwo’s remaking of the world system, I reimagine reparations for health in three parts. First, collective reparations which enable us to make links around the collectivist aspect of the right to health in where one’s individual rights are contingent on the rights of others thereby enabling global solidary arguments. This perspective is also particularly relevant within the context of the global south where there is a strong tradition of relational as opposed to individualistic rights.

Thinking of collective obligations enables us to think about the second part which would involve the transformation of systems through what I term transformative reparations, which is an evolving approach to address historical injustices, that goes beyond traditional forms of reparations such as compensation. This concept aims to provide redress for past wrongs and to change fundamentally the structures and systems that allowed those injustices to occur.^150^ Transformative reparations are future-oriented and enable systemic change. This approach is heavily advocated in feminist literature, as transformative reparations focus on providing redress by tackling the structural and socio-economic inequalities faced by affected communities, rather than merely providing monetary reparations, which would return them to a state of inequality, poverty, marginalisation, and discrimination.^151^ Therefore, providing transformative reparations (that takes into account impacts such as intergenerational trauma, unfavourable health outcomes linked to colonial medicines and looting of resources) such as fixing unequal trade relations between the Global South and North is the only just and equitable form of reparations.

The third phase of this approach is to take agency seriously. It is important to recognise the ways in which states from the Global South are reclaiming the global health space to articulate their demands. There are already demands in place by groups such as the Equity Group which is a group that was established to promote a fairer distribution of resources within the GHL system and has articulated its demands and what strategies they are using both in international negotiations but also outside them.^152^

### 4.1 Reparations: GHL potential or mirage?

Reparations necessitate moving beyond programs to structures through the idea of a new Pandemic Treaty. In the wake of the Covid-19-pandemic, the Director-General of the WHO established the Independent Panel for Pandemic Preparedness and Response which was tasked with initiating an independent and comprehensive evaluation process to review the devasting impact of the pandemic, particularly regarding vaccine inequality, and to propose a way forward.^153^ In 2021, WHO and its member States officially set up an intergovernmental negotiating body to draft and negotiate an international instrument for pandemic prevention, preparedness; commonly referred to as the Pandemic Treaty of Accord.^154^ The new treaty was intended to prioritise equity, both in the negotiating process and in its outcome.^155^ This focus on equity was particularly important as it underscored the paramount necessity of ensuring equitable access to vaccines, countermeasures, diagnostics, as well as fostering broader structural health system resilience through the promotion of Universal Health Coverage.

The Pandemic Accord, therefore, presented an opportunity to create structural changes to the international legal system to prevent the reproduction of systemic inequalities.^156^ The Africa Group’s strategy for the treaty negotiations involved uniting around common positions and presenting a cohesive stance on key issues to increase the likelihood of their perspectives to be acknowledged, considered, and incorporated. Their arguments were rooted in equity, and they were particularly vocal in three key areas. At the heart of their approach was the notion of African agency, which centres a post crisis view of what equity means to them within their of negotiations for the Pandemic Treaty.^157^ Working with the African Union, they have attempted to link vaccine manufacturing to a broader political project that has at the heart of it technology transfers so African countries can make the vaccines they need not only through pandemics but also ensure that they are sustainable. Additionally, there is an increasing realisation that in order for these vaccine manufacturing hubs to work in Africa, they will need to rely on broader South-South collaboration.^158^

### 4.2 Vaccine manufacturing and the role of technology transfers

There have been increasing calls to build the capacity for local manufacturing of vaccines, diagnostics, and therapeutics in Africa.^159^ The African Union has already pledged to increase the share of locally manufactured vaccines from 1% to 60% by 2040, an effort supported by the Global Vaccine Alliance’s recent establishment, African Vaccine Manufacturing Accelerator (AVMA). This initiative is set to provide up to one billion US dollars in funding to bolster vaccine manufacturing on the continent.^160^

At the heart of this initiative is the recognition that existing treaties, such as the TRIPS Agreement, must be challenged in order to engender a reparatory system. In the interim, incremental changes through a new Pandemic Accord could allow the provision of both direct and indirect incentives for technology transfer to ensure that countries in the Global South can exercise autonomy in manufacturing of routine medical products in order to enable the long-term survival of local vaccine manufacturing. Additionally, there have been calls for mandatory technology transfer during pandemics to ensure equitable access to essential medical products.^161^ One of the most promising approaches to facilitating technology transfer has been proposals to link it to research and development. These proposals suggest that pandemic funding mechanisms should provide multinational corporations with research and development funding in exchange for the compulsory transfer of technology, ensuring that innovations benefit a broader global population rather than being restricted by intellectual property barriers. ^162^

### 4.3 South-South cooperation

The promotion of South-South partnerships serves as a mechanism to disrupt reliance on vaccine donations from the Global North. Reparative justice seeks to rethink the more equitable and resilient global health responses by diversifying production locations from the global north to the south.^163^ Given the uneven dynamics, South-South partnerships are critical to the sustenance of this process. For instance, the Health Development Partnership for Africa and the Caribbean (HeDPAC), launched in December, 2023 is a collaboration by leaders from Rwanda, Guyana and Barbados which aimed to transfer knowledge aimed at strengthening health infrastructure between Africa and the Caribbean in vaccine manufacturing.^164^ HeDPAC aims to produce not only vaccines but also oncological drugs, and women’s health products in order to ensure that vaccine manufacturing is sustainable.^165^ As partnerships that involve South-South partnerships evolve over time, they aim to upend the colonial North/South dependencies that exist in Global Health.

## 5 Conclusion

At the moment, the fight for reparations feels abstract and highly speculative but as this article has illustrated there is a causal link between the harm of colonialism and the resulting lack of access to essential medicines. Symbolic and material reparations through changes to GHL structures are critical in order to create long term sustainable changes to these problems.

While the fight for reparations and its blueprint may currently seem abstract, it is rooted in a profound need to address the historical injustices of colonialism and their ongoing impact on access to medicines. Historical precedents, such as the reparations paid to Holocaust survivors and the acknowledgment of injustices faced by Indigenous populations, demonstrate that change is possible when there is a collective will to address past wrongs. Transformative reparations are vital to restructure global health systems, and it is the only way of fostering long-term sustainable change. The viability of these reparations, although challenged by practical concerns, such as political and legal resistance, is grounded in the hopeful pursuit of justice and equity that underpins international law. As the global community continues to grapple with the legacies of colonialism, the call for reparations must remain at the forefront of the discourse on global health equity.

## Footnotes

1 See eg Warwick Anderson, *Colonial Pathologies: American Tropical Medicine, Race, and Hygiene in the Philippines* (Duke University Press, Durham 2006) 2–4; Mark Harrison, *Medicine in an Age of Commerce and Empire: Britain and its Tropical Colonies 1660–1830* (OUP, Oxford 2011); Pratik Chakrabarti, *Medicine and Empire: 1600–1960* (Palgrave Macmillan Division of St Martin’s Press LLC, New York 2014) xiii–xv, 41–53.

2 Nandini Bhattacharya, *Contagion and Enclaves: Tropical Medicine in Colonial India* (Liverpool University Press, Liverpool 2012) 119; Helen Tilley, ‘Medicine, Empires, and Ethics in Colonial Africa’ (2016) 18 AMA Journal of Ethics 743, 746.

3 Agreement on Trade-Related Aspects of Intellectual Property Rights (adopted 15 April 1994, entered into force 1 January 1995) 1869 UNTS 299 (TRIPS Agreement).

4 UNGA, ‘Declaration of Commitment on HIV/AIDS’ (27 June 2001) UN Doc A/RES/S-26/2.

5 WTO, ‘Ministerial Declaration’ (14 November 2001) WTO Doc WT/MIN(01)/DEC/1; Carlos Correa and Duncan Matthews, *The Doha Declaration Ten Years on and Its Impact on Access to Medicines and the Right to Health*, UNDP Discussion Paper (2011); Carlos Correa, ‘Implementation of the WTO General Council Decision on Paragraph 6 of the Doha Declaration on the TRIPS Agreement and Public Health’ (Drug Action Programme of the Department of Essential Drugs and Medicines Policy, April 2004) <http://www.who.int/medicines/areas/policy/WTO_DOHA_DecisionPara6final.pdf> accessed 20 March 2025.

6 Nicholas Vincent, ‘TRIP-ing Up: The Failure of TRIPS Article 31bis’ (2020) Gonzaga Journal of International Law https://ssrn.com/abstract=3778945 accessed 20 March 2025.

7 Siobhán ES Murillo, ‘Fair or Fraud: Has the Protocol Amending TRIPS Flourished or Failed?’ (2017) 27 Indiana International and Comparative Law Review 191, 206ff.

8 Coleman Bazelon, Alberto Vargas, Rohan Janakiraman et al, ‘Report on Reparations for Transatlantic Chattel Slavery in the Americas and the Caribbean’ (Brattle, 2023) 49 <https://www.brattle.com/insights-events/publications/brattle-consultants-quantify-reparations-for-transatlantic-chattel-slavery-in-pro-bono-paper/> accessed 12 February 2025.

9 Ibid 49–78.

10 Ibid 17.

11 Audrey R Chapman and A Karim Ahmed, ‘Climate Justice, Humans Rights, and the Case for Reparations’ (2021) 23(2) Health and Human Rights 81; M Burkett, ‘Climate Reparations’ (2009) 10 Melbourne Journal of International Law 509; O Taiwo and B Cibralic, ‘The Case for Climate Reparations’ Foreign Policy (10 October 2020) <https://foreignpolicy.com/2020/10/10/case-for-climate-reparations-crisis-migration-refugees-inequality/> accessed 11 March 2025; M Sheller, ‘The Case for Climate Reparations’ Bulletin of the Atomic Scientists (6 November 2020); KK Perry, ‘Realizing Climate Reparations: Towards a Global Stabilization Fund and Resilience Fund Programme for Loss and Damage in Marginalized and Former Colonized Societies’ (1 March 2020) <https://papers.ssrn.com/sol3/papers.cfm?abstract_id=3561121> accessed 11 March 2025.

12 Margaret Urban Walker, ‘Making Reparations Possible: Theorizing Reparative Justice’ in Claudio Corradetti and Nir Eisikovits (eds), *Theorizing Transitional Justice* (Routledge, London 2015) 211, 216.

13 Ibid.

14 Ellen J Amster, ‘The Past, Present and Future of Race and Colonialism in Medicine’ (2022) 194 Canadian Medical Association Journal 708, 708–709.

15 Iona McCleery, ‘What is “Colonial” About *Medieval* Colonial Medicine? Iberian Health in Global Context’ (2015) 7(2) Journal of Medieval Iberian Studies 151, 153.

16 Olaniyi Bojuwoye, ‘Indigenous African Healing’ in Roy Moodley and Eunjung Lee (eds), *The Routledge International Handbook of Race, Culture and Mental Health* (1st edn, Routledge, London 2020) 497–500.

17 McCleery (n 15).

18 Bojuwoye (n 16).

19 Amster (n 14) 710.

20 Ibid.

21 Ibid.

22 Bhattacharya (n 2) 119; Tilley (n 2).

23 Ibid.

24 US Hospital Survey and Construction Act 1946 (Pub L 79–725, 60 Stat 1040).

25 Chin Hsien-Yu, ‘Colonial Medical Police and Postcolonial Medical Surveillance Systems in Taiwan, 1895–1950s’ (1998) 13 Osiris: Beyond Joseph Needham: Science, Technology, and Medicine in East and Southeast Asia 326.

26 Norman Howard-Jones, *The Scientific Background of the International Sanitary Conferences 1851–1938* (World Health Organization, Geneva 1975) 11.

27 See discussion on the background to the International Sanitary Conferences and resulting treaties from 1851 to 1938 in World Health Organization ‘International Sanitary Regulations: Proceedings of the Special Committee and of the Fourth World Health Assembly on WHO Regulations No. 2’ (1952) 1.

28 See International Sanitary Convention of 1892 (adopted 30 January 1892, entered into force 3 December 1892) UKTS 8/1893, art 4.

29 J’Nese Williams, ‘Public Health, Authority, and the Colonial State: Cholera in India’ (2021) Harvard Library Bulletin 3.

30 International Sanitary Convention of 1893 (adopted 15 April 1893, entered into force 1 June 1894) UKTS 4/1894, Title I.

31 Matiangai VS Sirleaf, ‘White Health and International Law’ (2 February 2024) University of Maryland Legal Studies Research Paper No 2024-03, 7 <https://papers.ssrn.com/abstract=4715089> accessed 12 February 2025.

32 See discussion on the savage-victim-saviour complex theorised in Makau wa Mutua, ‘Savages, Victims, and Saviors: The Metaphor of Human Rights’ (2001) 201, 202–204 <https://digitalcommons.law.buffalo.edu/journal_articles/570> accessed 10 February 2025.

33 Catherine Z Worsnop et al, ‘The Unintended Consequences of Information Provision: The World Health Organization and Border Restrictions during COVID-19’ (2023) 24 International Studies Perspectives 39, 45–46.

34 WHO, ‘COVID-19 Weekly Epidemiological Update: Data as Received by WHO from National Authorities, as of 27 December 2020, 10 am CET’ (WHO, 29 December 2020) <https://www.who.int/publications/m/item/weekly-epidemiological-update---29-december-2020> accessed 11 March 2025.

35 Sebastian Payne and Jim Pickard, ‘UK Exempts US from Travel Ban Despite High COVID-19 Case Rates’, *Financial Times* (London, 26 April 2020) <https://www.ft.com/content/4ab490c1-a0ad-453b-9c0f-c3c0f4fdaab2> accessed 11 March 2025.

36 Madalitso Z Phiri, ‘The Specter of Race in Global Covid-19 Responses: The Future Is Decolonial’ (2024) 31(2) Global Health Promotion 43, 46–47.

37 Alison Bashford, ‘Imperial Hygiene: A Critical History of Colonialism, Nationalism, and Public Health’ (Palgrave Macmillan, New York 2004) 84–87.

38 Joe Hernandez, ‘African Leaders Condemn Travel Restrictions as Omicron Variant Spreads Globally’, NPR (30 November 2021) <https://www.npr.org/sections/goatsandsoda/2021/11/30/1059780197/african-leaders-condemn-travel-restrictions-as-omicron-variant-spreads-globally> accessed 12 February 2025.

39 ‘Omicron COVID Variant Was in Europe before South African Scientists Detected and Flagged It to the World’, CBS News (30 November 2021) <https://www.cbsnews.com/news/omicron-variant-covid-in-europe-netherlands-before-alert-raised/> accessed 12 February 2025.

40 ‘Ebola Crisis: Ivory Coast Closes Land Borders’, BBC News (23 August 2014) <https://www.bbc.co.uk/news/world-africa-28913253> accessed 24 February 2025; Rebecca Kilberg, ‘Building Borders around Ebola’, Migration Policy Institute (16 December 2014) <https://www.migrationpolicy.org/article/building-borders-around-ebola> accessed 24 February 2025.

41 ‘Ebola’s Lost Blood: The Row over Samples Flown out of Africa as Big Pharma Set to Cash In’ (The Telegraph, 20 December 2019) <https://www.telegraph.co.uk/global-health/science-and-disease/ebolas-lost-blood-row-samples-flown-africa-big-pharma-set-cash/> accessed 11 March 2025.

42 BS Chimni, ‘The Past, Present and Future of International Law: A Critical Third World Approach’ (2007) 8 Melbourne Journal of International Law 499, 9, 12, 17.

43 Myriam Mertens, *Chemical Compounds in the Congo: Pharmaceuticals and the ‘Crossed History’ of Public Health in Belgian Africa (ca 1905-1939)* (Ghent University, Ghent 2014) 167, 206, 237.

44 Geoffrey Banda, Samuel Wangwe and Maureen Mackintosh, ‘Making Medicines in Africa: An Historical Political Economy Overview’ in Maureen Mackintosh et al (eds), *Making Medicines in Africa: The Political Economy of Industrializing for Local Health* (Palgrave Macmillan, London 2016) 10–11, 18.

45 Geoffrey Banda, ‘The Political Economy of the African Pharmaceutical Sector’s “Industrial Underdevelopment” Lock-in: The Importance of Understanding the Impact of Persistent Colonial Extractive Institutions’ (2023) 8 Frontiers in Research Metrics and Analytics 1, 3; Jessica Pourraz, ‘Making Medicines in Post-Colonial Ghana: State Policies, Technology Transfer and Pharmaceuticals Market’ (2022) 311 Social Science and Medicine 1, 3.

46 Pourraz (n 45) 3; Banda, Wangwe and Mackintosh (n 44) 8.

47 Pourraz (n 45) 4.

48 Banda, Wangwe and Mackintosh (n 37) 14.

49 Ibid 12.

50 Dani Rodrik, ‘Goodbye Washington Consensus, Hello Washington Confusion? A Review of the World Bank’s “Economic Growth in the 1990s: Learning from a Decade of Reform”‘ (2006) 44(4) Journal of Economic Literature 973..

51 Karen J Alter, ‘From Colonial to Multilateral International Law: A Global Capitalism and Law Investigation’ (2021) 3 International Journal of Constitutional Law 789, 839.

52 Anderson (n 1) 214.

53 Robert S Levine and others, ‘Tuskegee Redux: Evolution of Legal Mandates for Human Experimentation’ (2012) 23(4) Journal of Health Care for the Poor and Underserved 104..

54 Lisa M Lee, Kayte Spector-Bagdady, Maneesha Sakhuja, ‘Essential Cases in the Development of Public Health Ethics’ in Drue H Barrett et al (eds), *Public Health Ethics: Cases Spanning the Globe* (Springer, Cham 2016) 48.

55 Adriana Petryna, *When Experiments Travel: Clinical Trials and the Global Search for Human Subjects* (Princeton University Press, Princeton 2009) 90, 93, 96, 127–129.

56 Petryna (n 55) 89–138.

57 Ibid.

58 Johanna Crane, ‘Adverse Events and Placebo Effects: African Scientists, HIV, and Ethics in the “Global Health Sciences”‘ (2010) 40 Social Studies of Science 843, 844.

59 Amaka Vanni, *Patent Games in the Global South: Pharmaceutical Patent Law Making in Brazil, India and Nigeria* (Hart Publishing, Oxford 2021) 39–40.

60 Ibid 42, 45, 48.

61 Laurelyn Whitt, *Science, Colonialism, and Indigenous Peoples: The Cultural Politics of Law and Knowledge* (CUP, Cambridge 2009) 13, 157–178.

62 Amaka Vanni, ‘On Intellectual Property Rights, Access to Medicines and Vaccine Imperialism’, TWAILR: Reflections (5 March 2021) <https://twailr.com/on-intellectual-property-rights-access-to-medicines-and-vaccine-imperialism/> accessed 12 February 2025.

63 Andreas Papamichail, ‘Reinscribing Global Hierarchies: COVID–19, Racial Capitalism and the Liberal International Order’ (2023) 99 International Affairs 1673, 1675–1679; Vanni (n 50).

64 Edouard Mathieu, Hannah Ritchie, Lucas Rodés-Guirao et al, ‘Coronavirus Pandemic (COVID-19)’, Our World in Data (2020) <https://ourworldindata.org/covid-vaccinations> accessed 28 February 2023.

65 Sakshi Prasad, Abia Shahid, Edzel Lorraine F Co et al, ‘Vaccine Apartheid: The Separation of the World’s Poorest and Most Vulnerable and the Birth of Omicron’ (2022) 10 Therapeutic Advances in Vaccines and Immunotherapy, 1; E Tendayi Achiume, ‘UN Expert Urges States to End “Vaccine Apartheid”’, United Nations (14 June 2022) <https://www.ohchr.org/en/press-releases/2022/06/un-expert-urges-states-end-vaccine-apartheid> accessed 24 January 2023.

66 Daniel Opoku Acquah, ‘The Unusual Extension of Imperial Intellectual Property Laws to Colonies in Africa’ in P Sean Morris, *Intellectual Property and the Law of Nations, 1860*–*1920* (Brill, Leiden 2022) 291–331.

67 James Thuo Gathii, ‘The Doha Declaration on Trips and Public Health Under the Vienna Convention of the Law of Treaties’ (2002) 15 Harvard Journal of Law and Technology 292, 295.

68 ‘Seven Reasons the EU Is Wrong to Oppose the TRIPS Waiver’, Human Rights Watch (3 June 2021) <https://www.hrw.org/news/2021/06/03/seven-reasons-eu-wrong-oppose-trips-waiver> accessed 9 February 2024; Ashleigh Furlong, Sarah Anne Aarup and Samuel Horti, ‘Who Killed the COVID Vaccine Waiver?’ (POLITICO, 10 November 2022) <https://www.politico.eu/article/covid-vaccine-poor-countries-waiver-killed/> accessed 9 February 2024.

69 Austin Landis, ‘In a Historic Move, Colombia Bypasses Patent to Access HIV Drug’, Al Jazeera (13 September 2024) <https://www.aljazeera.com/features/longform/2024/9/13/in-a-historic-move-colombia-bypasses-a-patent-to-access-a-key-hiv-drug> accessed 13 February 2025.

70 Sarfaraz K Niazi, ‘Making COVID-19 mRNA Vaccines Accessible: Challenges Resolved’ (2022) 21 Expert Review of Vaccines 1163, 1173.

71 Siddhanth Sharma, Nisrine Kawa and Apoorva Gomber, ‘WHO’s Allocation Framework for COVAX: Is It Fair?’ (2021) 48 Journal of Medical Ethics 434; Ampson Hagan, ‘Coloniality of Waithood: Africa’s Wait for COVID-19 Vaccines amid COVAX and TRIPS’ (2023) 66 African Studies Review 327, 335.

72 Michael Penn, ‘If Rich Countries Don’t Share Their Vaccines, the Pandemic Could Stretch On For Years’ (Duke Global Health Institute, 24 February 2021) <https://globalhealth.duke.edu/news/if-rich-countries-dont-share-their-vaccines-pandemic-could-stretch-years> accessed 24 February 2025.

73 James Thuo Gathii, ‘Third World Perspectives on Global Pharmaceutical Access’ in Michael A Santoro and Thomas M Gorrie (eds), *Ethics and the Pharmaceutical Industry in the 21*^*st*^ *Century* (CUP, Cambridge 2005) 337–339.

74 Niazi (n 70) at 1166.

75 Sharifah Sekalala and Belinda Rawson, ‘Manufacturing Inequality: Examining the Racial-Capitalist Logics behind Global Pandemic Vaccine Production’, Afronomicslaw (23 January 2023) <https://www.afronomicslaw.org/category/analysis/manufacturing-inequality-examining-racial-capitalist-logics-behind-global> accessed 13 February 2025.

76 Achille Mbembe, ‘Necropolitics’ (2003) 15(1) Public Culture 11, 17, 23-27.

77 Rodger Bosch, ‘The mRNA Vaccine Technology Transfer Hub’, World Health Organization (21 June 2021) <https://www.who.int/initiatives/the-mrna-vaccine-technology-transfer-hub> accessed 24 February 2025.

78 Ntina Tzouvala, *Capitalism as Civilisation: A History of International Law* (CUP, Cambridge 2020) 161, 163; Sundhya Pahuja, *Decolonising International Law: Development, Economic Growth and the Politics of Universality* (CUP, Cambridge 2011) 45–47.

79 Antony Anghie, *Imperialism, Sovereignty and the Making of International Law* (CUP, Cambridge 2005) 38–39; Antony Anghie, ‘The Evolution of International Law: Colonial and Postcolonial Realities’ (2006) 27 Third World Quarterly 739.

80 For an analysis of the TRIPS negotiations and the specific factors shaping the development and design of the TRIPS Agreement see Susan K Sell, *Private Power, Public Law: The Globalization of Intellectual Property Rights* (CUP, Cambridge 2003) 1, 2; Emir Crowne, ‘Fishing TRIPS: A Look at the History of the Agreement on Trade-Related Aspects of Intellectual Property’ (17 December 2011) 2 Creighton International and Comparative Law Journal 77, 79–80.

81 Ibid.

82 Ibid.

83 Pahuja (n 78) 89–90.

84 See previous discussion on the historical foundations of pharmaceutical manufacturing in Africa; see generally Gathii (n 58).

85 James Thuo Gathii, ‘Approaches to Accessing Essential Medicine and the TRIPS Agreement’ in Peter K Yu (ed), *Intellectual Property and Information Wealth* (Greenwood Publishing Group, Westport 2006) 398–399; James Thuo Gathii, ‘Rights, Patents, Markets and the Global Aids Pandemic’ (2002) 14 Florida Journal of International Law 261, 278, 282.

86 ‘Reparation, n’ (Oxford English Dictionary, OUP) <https://www.oed.com/dictionary/reparation_n?tab=meaning_and_use#26108145> accessed 18 March 2025.

87 Margaret Urban Walker, *What Is Reparative Justice?* (Marquette University Press, Milwaukee 2010) 9–11.

88 Office of the High Commissioner for Human Rights, ‘Reparations: OHCHR and Transitional Justice’, United Nations<https://www.ohchr.org/en/transitional-justice/reparations> accessed 12 February 2025.

89 Matthew Evans and David Wilkins, ‘Transformative Justice, Reparations and Transatlantic Slavery’ (2019) 28 Social & Legal Studies 137, 137–157; see also Ta-Nehisi Coates, ‘The Case for Reparations’, The Atlantic (June 2014) <https://www.theatlantic.com/magazine/archive/2014/06/the-case-for-reparations/361631/> accessed 9 March 2025.

90 Federico Lenzerini, *Reparations for Indigenous Peoples: International and Comparative Perspectives* (OUP, Oxford 2008).

91 Elise Klein, ‘Beyond Compensation: Reparations and the Ongoing Colonization of Australia’ (2024) 55 Development and Change 830, 830–854.

92 Ellie Hamrick and Haley Duschinski, ‘Enduring Injustice: Memory Politics and Namibia’s Genocide Reparations Movement’ (2017) 11 Memory Studies 437, 437–454; see also Milica Kostić and Sandra Orlović, ‘Reparations for Victims of War within the Western Balkans EU Accession Negotiations: Serbia Case Study’ in Carla Ferstman, Mariana Goetz and Alan Stephens (eds), *Reparations for Victims of Genocide, War Crimes and Crimes against Humanity. Systems in Place and Systems in the Making* (Brill, Leiden 2009) 606–628.

93 Kirk W Junker, Saskia Münster and Mrinralini Shinde, ‘A Question of Trust: Building a Reparative Legal Regime in the Face of Climate-Induced Migration’ (2022) 52 Environmental Policy and Law 265, 265–276; see also Maxine Burkett, ‘Climate Reparations’ (2009) 10 Melbourne Journal of International Law 509, 509–542.

94 Pablo De Greiff, ‘Introduction Repairing the Past: Compensation for Victims of Human Rights Violations’ in Pablo De Greiff (ed), *The Handbook of Reparations* (OUP, Oxford 2006) 1–18; Pablo De Greiff, ‘Justice and Reparations’ in Pablo De Greiff (ed), *The Handbook of Reparations* (OUP, Oxford 2006) 454.

95 Regula Ludi, *Reparations for Nazi Victims in Postwar Europe* (CUP, Cambridge 2012) 2–31.

96 UNGA, ‘Basic Principles and Guidelines on the Right to a Remedy and Reparation for Victims of Gross Violations of International Human Rights Law and Serious Violations of International Humanitarian Law’ (16 December 2005) UN Doc A/RES/60/147 para 18.

97 UNGA, ‘Final Report on The Right of Everyone to the Enjoyment of the Highest Attainable Standard of Physical and Mental Health, Dainius Pūras’ (16 July 2020) UN Doc A/75/163 para 2.;CERD, ‘Statement on the Coronavirus (COVID-19) Pandemic and Its Implications under the International Convention on the Elimination of All Forms of Racial Discrimination’ (Hundred and first session, 4-7 August 2020) < https://www.ohchr.org/sites/default/files/Documents/HRBodies/TB/COVID19/Statement-CERD-COVID-19.docx#:~:text=Both%20with%20regard%20to%20the,Elimination%20of%20All%20Forms%20of> accessed 22 March 2025.

98 UNGA, ‘Report of the Special Rapporteur on contemporary forms of racism, racial discrimination, xenophobia and racial intolerance’ (21 August 2019) UN Doc A/74/321 para 26.

99 UNGA, ‘Report of the Working Group of Experts on People of African Descent’ (25 August 2021) UN Doc A/76/302, 19–20.

100 Frantz Fanon, *The Wretched of the Earth* (Constance Farrington tr, Penguin Modern Classics 2001) 1–3.

101 David C Gray, ‘A No-Excuse Approach to Transitional Justice: Reparations as Tools of Extraordinary Justice’ (2010) 87 Washington University Law Review 1043, 1050.

102 Alfred L Brophy, *Reparations Pro and Con* (OUP, New York 2006) 141.

103 For a discussion on the relationship between colonialism and the making of international law, see Anghie, *Imperialism, Sovereignty and the Making of International Law* (n 79).

104 Antony Anghie, ‘Rethinking International Law: A TWAIL Retrospective’ (2023) 34(1) European Journal of International Law 93, 93–97; James Thuo Gathii, ‘Imperialism, Colonialism and International Law’ (2007) 54 Buffalo Law Review 1013, 1013–1033.

105 See generally UNGA, ‘Report of the Working Group of Experts on People of African Descent’ (n 99).

106 Kaimipono David Wenger, ‘Causation and Attenuation in the Slavery Reparations Debate’ (2005) 40 University of San Francisco Law Review 279, 11–14; Eric K. Yamamoto and Susan K Serrano, ‘Reparations Theory and Practice Then and Now: Mau Mau Redress Litigation and the British High Court’ (2012) 18 Asian Pacific American Law Journal 71, 101.

107 Steven Wheatley, ‘Revisiting the Doctrine of Intertemporal Law’ (2021) 41 Oxford Journal of Legal Studies 484.

108 International Law Commission, ‘Draft Articles on Responsibility of States for Internationally Wrongful Acts’ (2001) UN Doc A/56/10, art 1.

109 Henning Grosse Ruse-Khan and Ashrutha Rai, ‘Intertemporality and Public Policy—Rethinking Temporal Conflict of Laws in Addressing Historic Injustices’ (4 November 2024) University of Cambridge Faculty of Law Research Paper No 34/2024 27–39 <https://papers.ssrn.com/abstract=5010122> accessed 12 February 2025.

110 *Legal Consequences of the Separation of the Chagos Archipelago from Mauritius in 1965* (Advisory Opinion) [2019] ICJ Rep 92.

111 See Wheatley (n 107).

112 *Moiwana Community v Suriname*, Judgment, Inter-American Court of Human Rights Series C No 124 (15 June 2005) para 43; UN Human Rights Committee, *Sandra Lovelace v Canada*, Communication No 24/1977 (30 July 1981) UN Doc CCPR/C/13/D/24/1977 para 11.

113 Nadia Bernaz, ‘Conceptualizing Corporate Accountability in International Law: Models for a Business and Human Rights Treaty’ (2021) 22(2) Human Rights Review 45, 46.

114 Peter J Dixon, ‘The Role of Reparations in the Transition from Violence to Peace’, *Oxford Research Encyclopedia of Politics* (2017) <http://politics.oxfordre.com/view/10.1093/acrefore/9780190228637.001.0001/acrefore-9780190228637-e-541> accessed 12 February 2025.

115 Truth and Reconciliation Commission, ‘Witness to Truth: Final Report of the TRC’ (Vol. 2, 2004) 192–197, 251–264, paras 482–512.

116 Ibid.

117 UNGA, ‘Report of the Working Group of Experts on People of African Descent’ (n 99) 18–19.

118 Ibid.

119 Benjamin Mason Meier, Thérèse Murphy, and Lawrence O Gostin, ‘The Birth and Development of Human Rights for Health’ in Lawrence O Gostin and Benjamin Mason Meier (eds), *Foundations of Global Health & Human Rights* (OUP, New York 2020) 23–25.

120 Sharifah Sekalala, Lisa Forman, Timothy Hodgson et al ‘Decolonising Human Rights: How Intellectual Property Laws Result in Unequal Access to the COVID-19 Vaccine’ (2021) 6(7) BMJ Global Health 1, 1–2; Sharifah Sekalala, Lisa Forman, Roojin Habibi et al, ‘Health and Human Rights Are Inextricably Linked in the COVID-19 Response’ (2020) 5(9) BMJ Global Health 1, 3.

121 Felix E Torres, ‘Economic and Social Rights, Reparations and the Aftermath of Widespread Violence: The African Human Rights System and Beyond’ (2021) 21 Human Rights Law Review 935, 936–938.

122 Ibid 938–943.

123 CESCR, ‘General Comment No 3: The Nature of State Parties’ Obligations (Art 2, Para 1, of the Covenant)’ (14 December 1990) UN Doc E/1991/23 para 7.

124 Judith Bueno De Mesquita, Claire Lougarre, Lisa Montel et al ‘Lodestar in the Time of Coronavirus? Interpreting International Obligations to Realise the Right to Health During the COVID-19 Pandemic’ (2022) 23 Human Rights Law Review 1, 15.

125 International Covenant on Economic, Social and Cultural Rights (adopted 16 December 1966, entered into force 3 January 1976) 993 UNTS 3, art 2.

126 CESCR, ‘Committee on Economic, Social and Cultural Rights: Statement on the Coronavirus Disease (COVID-19) Pandemic and Economic, Social and Cultural Rights’ (17 April 2020) UN Doc E/C.12/2020/1 para 15; CESCR, ‘Concluding Observations of the Committee on Economic, Social and Cultural Rights: Latvia’ (7 January 2008) UN Doc E/C.12/LVA/CO/1 paras 38-45; CESCR, ‘Committee on Economic, Social and Cultural Rights: Concluding Observations on the Seventh Periodic Report of Finland’ (30 March 2021) UN Doc E/C.12/FIN/CO/7 paras 8–9, 41–45; CESCR, ‘Concluding Observations on the Third Periodic Report of Kuwait’ (3 November 2021) UN Doc E/C.12/KWT/CO/3 para 40.

127 Elise Klein and Elizaveta Fouksman, ‘Reparations as a Rightful Share: From Universalism to Redress in Distributive Justice’ (2022) 53 Development and Change 31, 50–51.

128 UNGA, ‘Right of Everyone to the Enjoyment of the Highest Attainable Standard of Physical and Mental Health: Report of the Special Rapporteur on the Right of Everyone to the Enjoyment of the Highest Attainable Standard of Physical and Mental Health, Tlaleng Mofokeng’ (16 July 2021) UN Doc A/76/172 paras 6, 13.

129 Bueno De Mesquita et al (n 124) 2.

130 See generally UNGA, ‘Report of the Special Rapporteur on contemporary forms of racism, racial discrimination, xenophobia and racial intolerance’ (n 98); UNGA, ‘Report of the Working Group of Experts on People of African Descent’ (n 99).

131 ‘Rutte Apologises for Slave Trade: “Human Dignity was Trampled Underfoot’”’, *DutchNews.nl* (19 December 2022) <https://www.dutchnews.nl/2022/12/rutte-apologises-for-slave-trade-human-dignity-was-trampled-underfoot/> accessed 4 March 2025.

132 ‘Netherlands Slavery: Saying Sorry Leaves Dutch Divided’, *BBC News* (The Hague, 19 December 2022) <https://www.bbc.co.uk/news/world-europe-63993283> accessed 12 February 2025; Almaz Teffera, ‘Dutch Apology on Slavery Only First Step’ (*Human Rights* Watch, 15 December 2022) <https://www.hrw.org/news/2022/12/15/dutch-apology-slavery-only-first-step> accessed 12 February 2025.

133 ‘Germany’s President Apologises for Killings in Tanzania under Colonial Rule’, *Al Jazeera* (1 November 2023) <https://www.aljazeera.com/news/2023/11/1/german-president-apologises-for-colonial-crimes-in-tanzania> accessed 13 February 2025.

134 K Delali Adogla-Bessa and Sammi Wiafe, ‘Denmark Apologizes to Ghana for Role in Slave Trade’, Citi 97.3 FM - Relevant Radio. Always (24 November 2017) <https://citifmonline.com/2017/11/denmark-apologizes-to-ghana-for-role-in-slave-trade/> accessed 13 February 2025.

135 Jack Royston, ‘King Charles’ Colonialism “Sorrow” Is “Empty” without Apology, Reparations’, *Newsweek* (23 November 2022) <https://www.newsweek.com/king-charles-colonialism-sorrow-empty-without-apology-reparations-south-africa-state-visit-1761674> accessed 13 February 2025.

136 Sean Coughlan, ‘No UK Apology over Slavery at Commonwealth Summit’, *BBC News* (18 October 2024) <https://www.bbc.com/news/articles/c0qzkg0ldqzo> accessed 13 February 2025.

137 Philip Oltermann, ‘Germany Agrees to Pay Namibia €1.1bn over Historical Herero-Nama Genocide’, *The Guardian* (Berlin, 28 May 2021) <https://www.theguardian.com/world/2021/may/28/germany-agrees-to-pay-namibia-11bn-over-historical-herero-nama-genocide> accessed 12 February 2025.

138 Ibid.

139 Derek Ross Soled, Avik Chatterjee, Daniele Olveczky et al, ‘The Case for Health Reparations’ (2021) 9 Frontiers in Public Health 1, 2–3; Alexandre Rotondo-Medina, ‘Reparations for Black Health’ (2022) 11 Journal of Race, Gender, and Ethnicity 215, 219–21; Eugene T Richardson, Momin M Malik, William A Darity Jr et al, ‘Reparations for Black American Descendants of Persons Enslaved in the U.S. and Their Potential Impact on SARS-CoV-2 Transmission’ (2021) Social Sciences and Medicine 1, 2.

140 Rotondo-Medina (n 139) 217.

141 Kristen Paige Green, ‘Letters to Soleil: Reproductive Reparations as Black Maternal Justice’ (2024) 112 Georgetown Law Journal 1543, 1546.

142 Robert B Baker, ‘The American Medical Association and Race’ (2014) 16 AMA Journal of Ethics 479.

143 David Bollier, ‘Black Commons, Community Land Trusts, and Reparations’, resilience (22 July 2020) #x003C;https://www.resilience.org/stories/2020-07-22/black-commons-community-land-trusts-and-reparations/> accessed 14 February 2025.

144 Gray (n 101) 1050.

145 Olúfémi O Táíwò, *Reconsidering Reparations* (OUP, New York 2022) 74.

146 Ibid 3.

147 Ibid 74.

148 This is a challenge that many TWAIL scholars grapple with. See for instance China Miéville, ‘The Commodity-Form Theory of International Law: An Introduction’ (2004) 17 Leiden Journal of International Law 271, 301; China Miéville, *Between Equal Rights: A Marxist Theory of International Law* (Brill, Leiden 2005) 295–320; Luis Eslava and Sundhya Pahuja, ‘Beyond the (Post)Colonial: TWAIL and the Everyday life of International Law’ (2012) 45 Verfassung In Recht und Übersee 195, 11–17.

149 Eslava et al (n 148) 210–212, 215.

150 Ruth Rubio-Marín, ‘The Gender of Reparations in Transitional Societies’ in Ruth Rubio-Marín (ed), *The Gender of Reparations: Unsettling Sexual Hierarchies While Redressing Human Rights Violations* (CUP, Cambridge 2009) 70; Rodrigo Uprimny Yepes, ‘Transformative Reparations of Massive Gross Human Rights Violations: Between Corrective and Distributive Justice’ (2009) 27 Netherlands Quarterly of Human Rights 625, 637–639.

151 Marín (n 150) 639.

152 Priti Patnaik, ‘May 2024 Deadline Conditional on Meeting Demands on Equity: 29 Member States of the Equity Group’, Geneva Health Files (3 November 2023) <https://genevahealthfiles.substack.com/p/may-2024-deadline-conditional-on> accessed 24 February 2025.

153 Helen Clark, Ellen Johnson Sirleaf, Mauricio Cárdenas et al, ‘COVID-19: Make it the Last Pandemic: The Independent Panel for Pandemic Preparedness & Response’ (2021) 8 <https://theindependentpanel.org/wp-content/uploads/2021/05/COVID-19-Make-it-the-Last-Pandemic_final.pdf> accessed 20 March 2025.

154 WHO, ‘World Health Assembly Agrees to Launch Process to Develop Historic Global Accord on Pandemic Prevention, Preparedness and Response’, WHO (1 December 2021) <https://www.who.int/news/item/01-12-2021-world-health-assembly-agrees-to-launch-process-to-develop-historic-global-accord-on-pandemic-prevention-preparedness-and-response> accessed 24 February 2025.

155 Ibid.

156 Ibid.

157 ‘Africa Speaks with One Voice on the Pandemic Agreement’, AfricaCDC (2 May 2024) <https://africacdc.org/news-item/africa-speaks-with-one-voice-on-the-pandemic-agreement/> accessed 13 February 2025.

158 At the time of writing there is no agreement on the Pandemic Accord, with negotiations still ongoing. The approach that African countries took is still a legitimate first step in the recognition of African agency within international treaty negotiations.

159 Abdulrahman A Saied, Asmaa A Metwally, Manish Dhawan et al, ‘Strengthening Vaccines and Medicines Manufacturing Capabilities in Africa: Challenges and Perspectives’ (2022) 14 EMBO Molecular Medicine 1.

160 ‘African Vaccine Manufacturing Accelerator (AVMA)’ (Gavi, the Vaccine Alliance, 2024) <https://www.gavi.org/programmes-impact/types-support/regional-manufacturing-strategy/avma> accessed 11 March 2025.

161 Acquah (n 66) 291.

162 WHO, ‘Intellectual Property Rights and Vaccines in Developing Countries: Meeting Report 19–20 April 2004’ (September 2004) WHO/IVB/04.21, 34–40.

163 Ibid.

164 WHO, ‘New South-South Health Cooperation Initiative Launched Linked Africa and the Caribbean’, WHO (19 December 2023) <https://www.who.int/news/item/19-12-2023-new-south-south-health-cooperation-initiative-launched-linking-africa-and-the-caribbean> accessed 14 February 2025.

165 Ibid.

